# Increased Circulating VAP-1 Levels Are Associated with Liver Fibrosis in Chronic Hepatitis C Infection

**DOI:** 10.3390/jcm8010103

**Published:** 2019-01-17

**Authors:** Marcel Kraemer, Marcin Krawczyk, Fozia Noor, Frank Grünhage, Frank Lammert, Jochen G. Schneider

**Affiliations:** 1 Department of Internal Medicine II, Saarland University Medical Center, 66421 Homburg, Germany; marcel.kraemer@mail.de (M.K.); marcin.krawczyk@uks.eu (M.K.); frank.gruenhage@kkh-ne.de (F.G.); frank.lammert@uks.eu (F.L.); 2 Laboratory of Metabolic Liver Diseases, Centre for Preclinical Research, Department of General, Transplant and Liver Surgery, Medical University of Warsaw, 02-097 Warsaw, Poland; 3 Luxembourg Centre for Systems Biomedicine (LCSB), University of Luxembourg, L-4367 Belvaux, Luxembourg; fozia.noor@uni.lu; 4 Department of Gastroenterology and Oncology, Rhein-Kreis-Neuss Hospital, 41515 Grevenbroich, Germany; 5 Centre Hospitalier Emile Mayrisch, L-4240 Esch-sur-Alzette, Luxembourg

**Keywords:** VAP-1, vascular adhesion protein 1, SSAO activity, semicarbazide-sensitive amino oxidase, chronic liver diseases, fibrosis, liver stiffness, Fibroscan, HCV

## Abstract

Vascular adhesion protein-1 (VAP-1) is a multifunction protein. While membrane-bound VAP-1 is an adhesion protein, soluble VAP-1 catalyzes the deamination of primary amines through its semicarbazide-sensitive amino oxidase (SSAO) activity. VAP-1 supports the transmigration of leukocytes and increases oxidative stress. In chronic liver diseases, it plays a role in leukocyte infiltration and fibrogenesis. Here, we measured VAP-1 plasma concentration and its SSAO activity in 322 patients with chronic hepatitis C infection and evaluated the association of VAP-1 with fibrosis stages. VAP-1 concentration strongly correlated with liver stiffness and was the second strongest influencing variable after gamma-glutamytransferase (GGT) for liver stiffness in regression analysis. The VAP-1 concentration increased with advancing fibrosis stages and the highest concentrations were found in patients with cirrhosis. According to the receiver operating characteristic (ROC) analysis, a VAP-1 cut-off value of 541 ng/mL predicted histologically confirmed cirrhosis (sensitivity 74%; specificity 72%). SSAO activity correlated only moderately with liver stiffness, showing a relatively small increase in advanced fibrosis. To our knowledge, this is the first study on VAP-1 in chronic hepatitis C infection showing its association with progressive fibrosis. In conclusion, VAP-1 plasma concentration, rather than its SSAO activity, may represent a non-invasive biomarker for monitoring fibrogenesis in patients with chronic hepatitis C infection.

## 1. Introduction

Vascular adhesion protein-1 (VAP-1) is a 170 kDa sialoglycoprotein. It exists in soluble and membrane-bound forms [1]. VAP-1 has an enzymatic domain that is responsible for its primary amine oxidase activity, also referred to as the semicarbazide-sensitive amino oxidase (SSAO) activity [2]. It is involved in the conversion of exogenous and endogenous amines, for example benzylamine and methylamine, into aldehydes by oxidative deamination, thereby releasing cytotoxic products such as hydrogen peroxide and ammonia. These cytotoxic products lead to increased oxidative stress and the formation of advanced glycation endproducts [3]. On the other hand, VAP-1 non-enzymatically triggers inflammation via an adhesion domain by attracting and supporting the adhesion and transmigration of leukocytes from vessels into the inflamed sites [4,5]. VAP-1 is stored in intracellular vesicles and is present on the membranes of the endothelial cells, muscle cells, adipocytes and hepatic sinusoidal endothelium. Under physiological conditions, the highest amount of membrane-bound VAP-1 exists on the endothelial cells of the lymph nodes [6]. In the soluble form, VAP-1 is present in the human blood [1].

VAP-1 concentration and, partly its SSAO activity, are modified in various pathological conditions, such as atherosclerosis [7], chronic kidney injury [8] and diabetes mellitus [9]. In chronic diseases with leukocyte infiltration, a considerable amount of VAP-1 is detectable on the endothelial cell surface of the affected tissue. In chronic liver diseases (CLDs), for example, VAP-1 is present on the hepatic sinusoidal endothelium and hepatic stellate cells [5,10,11].

The serum VAP-1 concentration differs between various CLDs, for example, patients with alcoholic liver diseases (ALDs) have higher VAP-1 concentrations when compared to those with primary biliary cholangitis and primary sclerosing cholangitis [6]. Moreover, significantly increased VAP-1 concentrations were detected in non-alcoholic fatty liver disease (NAFLD) patients in comparison to a matched metabolic syndrome group [5]. Notably, VAP-1 concentration allowed distinction between non-alcoholic steatohepatitis (NASH) and simple steatosis. Like other CLDs, chronic hepatitis C (HCV) infection also results in fibrosis that may lead to the cirrhosis of the liver. According to the World Health Organization (WHO), there are an estimated 71 million chronic HCV infected people in the world [12]. Despite the availability of novel direct acting antiviral therapies, HCV infection is still the most common cause of cirrhosis worldwide [12].

Here, we studied the concentration and the activity of plasma VAP-1 in patients with chronic HCV infection. We investigated the levels of VAP-1 and SSAO activity in different fibrosis stages, which were assessed by transient elastography (Fibroscan^®^) as liver stiffness and liver biopsy in some cases. We aimed to discern diagnostic patterns and to estimate the informative value of measuring VAP-1 concentration and activity as markers for fibrosis severity.

## 2. Experimental Section

This study was conducted on a subgroup of a study cohort consisting of 899 patients with different CLDs [13]. From this cohort, we selected 538 subjects suffering from chronic HCV infection. Depending on the availability of frozen blood samples and clinical parameters, 322 patients were considered eligible for our study (Supplemental Figure S1). All patients gave written informed consent. The study was approved by the local ethical committee (approval number of Ärztekammer des Saarlandes, Germany: 271/11).

The liver stiffness of all 322 patients was measured by transient elastography (Fibroscan^®^, Echosens SA, Paris, France) [13] as a non-invasive measurement of liver stiffness. We divided the patients according to the fibrosis stages using the cut-off values from Castera et al. [14]; no/mild fibrosis corresponds with stage F0/F1 (≤7.0 kPa), moderate fibrosis with F2/F3 (7.1–12.4 kPa), and severe fibrosis with F4 (≥12.5 kPa). A liver biopsy using the Menghini technique [15] with a 1.8-mm needle was carried out in 92 patients. The staging was performed according to the classification of Desmet et al. [16]. Overall, 38 cases were diagnosed with liver cirrhosis (fibrosis stage F4) according to the biopsy results. 

The AST to platelet ratio index (APRI) and Forns index for the assessment of liver fibrosis in chronic HCV patients could only be determined in 144 and 138 patients respectively, due to missing laboratory values for other patients.

Plasma concentration of VAP-1 was determined using an enzyme-linked immunoassay (ELISA) from R&D Systems (Minneapolis, MN, USA). According to the manufacturer, the intra-assay variation was 2.1%/1.5%/2.4%, while the inter-assay variation was 4.5%/4.8%/4.7% in three independent experiments. 

We measured the SSAO activity using an Amplex ^®^ Red Monoamine Oxidase assay kit from Invitrogen™ Molecular Probes™ (ThermoFisher Scientific, Waltham, MA, USA). SSAO activity was determined by a fluorometric detection of hydrogen peroxide produced by different amino oxidases, including the semicarbazide-sensitive amino oxidase (SSAO, the enzymatic domain of VAP-1) after adding benzylamine as a substrate. Each sample was measured with and without the specific SSAO inhibitor semicarbazide and the specific activity was calculated by subtraction. 

Statistical analysis was performed with SPSS 22.0 (IBM, Armonk, NY, USA). According to the Kolmogorov–Smirnov test, the VAP-1 concentration and its activity were not normally distributed. Hence, after log transformation, we used Mann–Whitney U test for the comparison of medians or ANOVA followed by Bonferroni post hoc test for multiple comparisons. Nonparametric Spearman correlation coefficient was used for non-normal data. Linear regression analysis was used to test for independent associations and clusters were compared with the Kruskal–Wallis test. For the determination of cut-off values and the calculation of the associated sensitivity and specificity, we used receiver operating characteristic (ROC) curve analysis. We use boxplots to present the results, and the dots represent the outliers. Results were considered significant at *p* < 0.05. The effect size is given by the beta values.

## 3. Results

Table 1 summarizes the baseline patient characteristics. The study cohort involved 322 patients (98 women and 224 men). The mean age was 49.7 (±12.1) years and the mean body mass index (BMI) was 24.3 (±4.6) kg/m². The liver stiffness values were not consistent with biopsy results in three patients with liver biopsy-confirmed cirrhosis. Moreover, nine patients were above the general liver stiffness cut-off value for cirrhosis (12.5 kPa), but were classified with stage F0–F3 fibrosis on the basis of the biopsy results. However, in 87% of the cases, liver stiffness and biopsy results were concordant. Overall, 106 patients had values higher than the cut-off for cirrhosis, whereas 216 patients had liver stiffness values less than 12.5 kPa. 

The patients with liver stiffness of ≥12.5 kPa, presumably having cirrhosis, had significantly higher plasma VAP-1 concentrations as compared to the patients with liver stiffness values less than 12.4 kPa and without cirrhosis (no/mild and moderate fibrosis). Similarly, the SSAO activity in these patients was significantly higher in comparison to patients with no/mild fibrosis (Table 1). The glutamic oxaloacetic transaminase (GOT) and gamma-glutamytransferase (GGT) activities, as well as bilirubin and thrombocytes, also differed significantly between patients with or without severe fibrosis/cirrhosis. The APRI and Forns index significantly increased with liver stiffness (Table 1).

**Figure 1:**
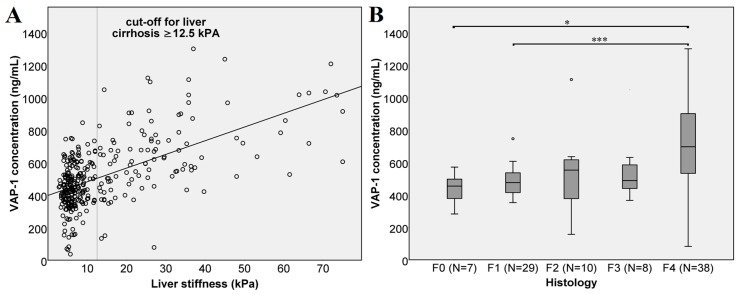
Vascular adhesion protein-1 (VAP-1) concentration and liver fibrosis. (**A**) VAP-1 concentration and liver stiffness of all 322 patients. The cut-off value for liver cirrhosis was set at ≥12.5 kPa. The line shows the trend of the VAP-1 concentration with increasing liver stiffness as assessed by transient elastography; (**B**) VAP-1 concentration in different fibrosis stages as assessed by biopsy-based histology. According to the Kruskal–Wallis test, there were no significant differences (*p* > 0.05) between different biopsy-based fibrosis stages (F0 vs. F1, F2 or F3; F1 vs. F2 or F3 and F2 vs. F3). There were significant differences between F0 vs. F4 (*p* = 0.012) and highly significant between F1 vs. F4 (*p* < 0.001). Significance is shown as * *p* < 0.05, and *** *p* < 0.001.

A comparison of VAP-1 concentrations and liver stiffness measurements (Figure 1A) shows a strong linear correlation (*r* = 0.528 Spearman, *p* < 0.0001). Likewise, we compared VAP-1 concentrations in patients classified in different fibrosis stages according to the biopsy results (Figure 1B). Here, the VAP-1 concentration did not differ between the individual fibrosis stages. We attribute this, at least in part, to the low number of biopsies in the individual groups (F0: *N* = 7, F2: *N* = 10, F3: *N* = 8). Nevertheless, severe fibrosis, stage F4 indicating cirrhosis, showed highly significant difference from mild (F1) fibrosis with *p* < 0.001 and significant difference from patients without fibrosis (F0) with a *p* = 0.012.

The VAP-1 concentration also significantly correlated with glutamate pyruvate transaminase (GPT), GOT, GOT/GPT ratio, GGT, cholesterol, bilirubin, albumin, platelets and age (Table 2). The SSAO activity showed a lower but significant, correlation with these parameters.

Based on these results, we performed a linear regression analysis to test for independent predictors for liver stiffness or fibrosis severity. Here, we included all the parameters which correlated with VAP-1 concentration and SSAO activity in the regression analysis: These were namely; GOT, GPT, GGT, cholesterol, bilirubin, platelets, age, weight, soluble fms-like tyrosine kinase-1 (sFlt1), high-sensitivity troponin T (hsTnT), endoglin, pro brain natriuretic peptide (proBNP), APRI and Forns index. In this analysis, VAP-1 concentration was the second strongest variable associated with liver stiffness after GGT (VAP-1: beta = 0.197, T score = 2.451, significance *p* = 0.016). When including the recently identified surrogate markers [17], the growth/differentiation factor 15 (GDF15), hepatocyte growth factor (HGF) and placental growth factor (PLGF); in the model, VAP-1 was still the second strongest influencing variable (VAP-1: beta 0.185, T score = 2.218, *p* = 0.029, Table 3). The influence of SSAO activity was less pronounced and was excluded in a stepwise procedure. 

**Figure 2:**
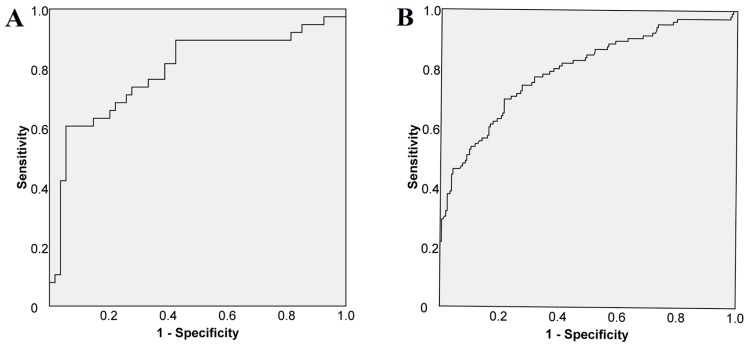
Receiver operating curve (**A**) to predict histologically confirmed cirrhosis (*N* = 92): sensitivity 74%; specificity 72%; area under the curve (AUC) 0.791, and (**B**) to predict liver stiffness cut-off for cirrhosis (*N* = 322): sensitivity 75%; specificity 73%; AUC 0.799.

We performed a ROC curve analysis to assess the cut-off value for VAP-1 in all 322 patients. The analysis demonstrated that a cut-off of 541 ng/mL of VAP-1 predicted histologically confirmed cirrhosis (in 92 patients) with a sensitivity of 74% and a specificity of 72% (Figure 2A). As for liver stiffness, a cut-off value ≥ 12.5 kPa predicted cirrhosis with a sensitivity of 75% and a specificity of 73% (Figure 2B).

**Figure 3:**
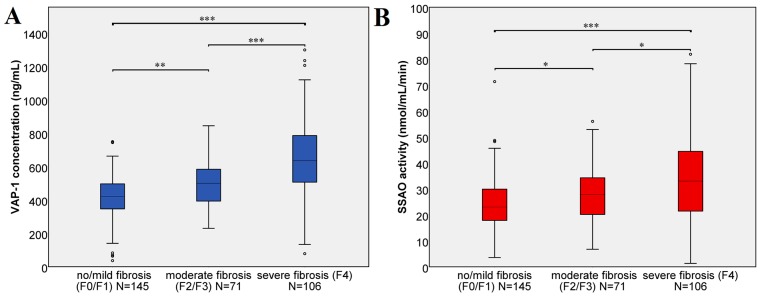
VAP-1 concentration and SSAO activity in mild, moderate and severe fibrosis. The classification is based on the liver stiffness. (**A**) VAP-I concentration. According to Kruskal–Wallis test, there were significant differences between no/mild fibrosis vs. moderate fibrosis (*p* = 0.002 **), no/mild fibrosis vs. severe fibrosis (*p* < 0.001 ***), moderate fibrosis vs. severe fibrosis (*p* < 0.001 ***) (**B**) SSAO activity. According to Kruskal–Wallis test, there were significant differences between no/mild fibrosis vs. moderate fibrosis (*p* = 0.023 *), no/mild fibrosis vs. severe fibrosis (*p* < 0.001 ***), moderate fibrosis vs. severe fibrosis (*p* = 0.028 *).

The biopsy results indicated significantly higher VAP-1 concentrations in patients with cirrhosis (*N* = 92, no cirrhosis vs. cirrhosis with a *p* < 0.0001, Supplemental Figure S2). The classification of the patients in mild (45%), moderate (22%) and severe fibrosis (33%) based on liver stiffness showed that the VAP-1 concentration increased with fibrosis severity (Figure 3A). Both mild fibrosis in comparison to moderate fibrosis and moderate fibrosis in comparison to severe fibrosis or cirrhosis was differentiated according to the VAP-1 concentration (*p* = 0.003 and *p* < 0.001 respectively).

In addition to its concentration, we measured the SSAO activity of VAP-1 in all patients. Although we observed a strong correlation with the VAP-1 concentration (Table 2) the SSAO activity of VAP-1 correlated only moderately with liver, APRI and Forns-index (Table 2). The SSAO activity of VAP-1 significantly increased in advanced fibrosis stage (F4) in comparison to no/mild fibrosis with a *p* < 0.001 as shown in Figure 3B. Significant differences were also observed between no/mild and moderate fibrosis (*p* = 0.023), as well as between moderate and severe fibrosis (*p* = 0.028). 

In the investigated cohort, eight patients suffered from hepatocellular carcinoma (HCC). The VAP-1 concentration in these patients was higher than the VAP-1 concentration in patients with liver cirrhosis but without HCC, albeit this difference was not significant (*p* > 0.05). 

## 4. Discussion

Varying concentrations of VAP-1 have been associated with CLDs indicating its possible role in the pathophysiology of these diseases. In a recent study, VAP-1 concentration was significantly higher in patients with NAFLD and NASH as compared to the patients with metabolic syndrome [5]. Based on these observations and others [18], VAP-1 was suggested as a potential therapeutic target in NAFLD, and possibly in other CLDs.

In our study on 322 chronic HCV infection patients, we show that the VAP-1 concentration, as well as the SSAO activity, were significantly elevated in patients with moderate or severe fibrosis especially in the presence of cirrhosis. Fibrosis stages were determined from the liver stiffness as measured by transient elastography. 

A direct comparison of the VAP-1 values with the previously reported changes in NAFLD [5] is not appropriate, yet our study indicates a stronger correlation of the VAP-1 concentration with GOT, GPT, GGT, bilirubin, cholesterol, platelets and albumin as parameters of liver injury and liver function. Furthermore, in our study, the VAP-1 concentration showed higher correlation coefficients for the fibrosis stage in chronic hepatitis C infection than those reported for NAFLD and NASH. Notably, our cohort included proportionally many more cirrhosis patients. The strong association between VAP-1 concentration and other laboratory parameters is likely due to our larger study cohort, which included patients in different fibrosis stages. The previous study on NAFLD and NASH patients reported increased VAP-1 concentration in fibrosis stage F ≥ 2 in comparison to F0/F1, whereas our study shows a stronger correlation with progressive fibrosis. The VAP-1 concentration significantly increased from mild to moderate and severe fibrosis. As the plasma VAP-1 concentration in CLDs is higher in hepatic veins than in portal veins, and furthermore, the membrane-bound VAP-1 concentration is elevated in hepatic stellate cells in increasing fibrosis [10], we assume that the affected liver parenchyma is the main source of the elevated plasma VAP-1 in chronic hepatitis C infection as well.

According to previous studies, VAP-1 supports leukocyte transmigration in the inflamed tissue and the deleterious effects of its SSAO activity lead to enhanced production of extracellular matrix proteins and increased oxidative stress. This process contributes to progressive fibrosis that ultimately leads to cirrhosis. Experimental studies showed a reduction of inflammation and fibrosis in VAP-1 deficient mice or in the presence of an SSAO inhibitor. We evaluated the SSAO activity in our cohort to assess its correlation with progressive fibrosis. However, in our study, the increase in SSAO activity was only moderate as compared to the increase in VAP-1 concentration. The SSAO activity correlated less with the markers of liver damage and did not allow an estimation of the progression of fibrosis. However, the difference between no/mild fibrosis vs. severe fibrosis/cirrhosis was highly significant. Possibly, the SSAO activity may no longer play a significant role when moderate and advanced fibrosis stages are reached.

In multivariate analysis, VAP-1 was an independent predictor of fibrosis stages and was the second strongest predicting variable after GGT. Our group has previously shown the predictive value of three serum markers, namely PLGF, HGF and GDF-15, for liver stiffness and fibrosis stages. Even when these parameters were included into the regression analysis, VAP-1 remained the second strongest independent predictor of fibrosis suggesting its major role in the disease progression. 

The significantly increased VAP-1 levels in the presence of cirrhosis allowed us to define a cut-off value for cirrhosis. For cirrhosis, we calculated a cut-off of 541 ng/mL of VAP-1 based on the biopsy results of 92 patients and a cut-off from the liver stiffness of more than 12.5 kPa in all 322 patients. These showed nearly the same predictive value. Furthermore, the non-invasive classification of the fibrosis progression into mild, moderate and severe fibrosis demonstrated that the VAP-1 concentration is also clearly elevated with moderate fibrosis. Presumably, VAP-1 levels continuously increase with sustained injury to the liver parenchyma. Thus, VAP-1 concentration may represent a non-invasive biomarker for progressive fibrosis and cirrhosis. Of note, in our study, we not only had liver stiffness measurements for all chronic HCV infection patients, but we also had liver biopsy results in more than a quarter of the cases. Additionally, we compared VAP-1 concentration and SSAO activity in all patients. On the other hand, we cannot exclude a selection bias, as not all participants of the original cohort with other CLDs could be included in our study. However, VAP-1 concentration differs between individual CLDs, thus further disease-specific studies are necessary. In future studies, more HCC patients should also be included to assess an increase of VAP-1 concentrations in patients who develop liver cancer.

## 5. Conclusions

To our knowledge, this is the first study on VAP-1 in chronic hepatitis C infection showing a direct association between VAP-1 plasma concentration and fibrosis severity. VAP-1 concentration increased with the severity of fibrosis and was significantly elevated in patients with cirrhosis. Although the SSAO activity of VAP-1 increased, the differences among the individual stages of fibrosis severity were only partly significant. Therefore, we conclude that VAP-1 concentration, rather than its SSAO activity, may represent a tool for monitoring fibrogenesis in the follow-up of patients with CLDs. 

## Figures and Tables

**Table 1:** Characteristics of the study cohort. The classification between no/mild, moderate and severe fibrosis is based on liver stiffness as assessed by transient elastography (Fibroscan^®^). No/mild fibrosis ≤7.1 kPa; moderate fibrosis 7.1–12.4 kPa; severe fibrosis ≥12.5 kPa. The *N*, the average and the *p* values for the differentiation between the fibrosis stages are given. Statistical analysis was performed with ANOVA followed by the Bonferroni post-hoc test. Significance is given as * *p* < 0.05, ** *p* < 0.01, and *** *p* < 0.001.

	*N*	Average			*p* Value		
		No/Mild Fibrosis	Moderate Fibrosis	Severe Fibrosis	No/Mild vs. Moderate Fibrosis	No/Mild vs. Severe Fibrosis	Moderatevs. Severe Fibrosis
Sex (1 = male, 2 = female)	322	1.34	1.28	1.26	1.00	0.515	1.00
Age (years)	322	46.90	50.92	52.74	0.113	<0.001 ***	0.574
Body mass index (BMI) in kg/m²	302	23.76	25.06	24.72	1.00	0.33	1.00
Liver stiffness (kPa)	322	5.26	9.05	29.81	<0.001 ***	<0.001 ***	<0.001 ***
Plasma VAP-1 (vascular adhesion protein-1) concentration in ng/mL	322	421.01	495.15	662.96	0.002 **	<0.001 ***	<0.001 ***
SSAO (semicarbazide-sensitive amino oxidase) activity in ng/mL/min)	317	24.49	28.18	34.86	0.148	<0.001 ***	0.312
APRI (AST to platelet ratio index)	144	0.21	0.33	1.05	0.013 *	<0.001 ***	<0.001 ***
Forns index	138	7.17	8.15	10.34	0.017 *	<0.001 ***	<0.001 ***
Platelet count (1000/µL)	308	226.19	201.70	134.65	0.154	<0.001 ***	<0.001 ***
Cholesterol (mg/dL)	223	171.07	167.26	153.86	1.00	0.111	0.301
GGT (gamma-glutamytransferase) in U/I	258	57.12	90.61	182.24	<0.001 ***	<0.001 ***	<0.001 ***
GPT (glutamate pyruvate transaminase) in U/I	257	61.36	90.66	122.00	0.003 **	<0.001 ***	1.00
GOT (glutamic oxaloacetic transaminase) in U/I	254	40.13	56.25	103.06	<0.001 ***	<0.001 ***	<0.001 ***
Bilirubin (mg/dL)	255	0.67	0.68	1.16	1.00	<0.001 ***	<0.001 ***

**Table 2:** Correlation analysis of VAP-1 concentration and semicarbazide-sensitive amino oxidase (SSAO) activity with standard laboratory parameters. Spearman correlating coefficients (r) and *p* values are shown. Significance is given as * *p* < 0.05, ** *p* < 0.01, and *** *p* < 0.001.

	VAP-1 Concentration		SSAO Activity	
	*r*	*p* Value	*r*	*p* Value
Fibrosis stage	0.513	<0.001 ***	0.233	0.026 *
Age	0.294	<0.001 ***	0.114	0.043 *
Platelets	−0.357	<0.001 ***	−0.241	<0.001 ***
Albumin	−0.460	<0.001 ***	−0.261	<0.001 ***
GOT/GPT	0.636	<0.001 ***	0.530	0.002 **
GOT	0.460	<0.001 ***	0.279	<0.001 ***
GPT	0.255	<0.001 ***	0.209	<0.001 ***
GGT	0.327	<0.001 ***	0.156	0.013 *
Bilirubin	0.158	0.011 *	0.128	0.042 *
Cholesterol	−0.171	0.010 *	−0.143	0.035 *
Liver stiffness	0.528	<0.001 ***	0.347	<0.001 ***
APRI	0.474	<0.001 ***	0.211	0.011 *
Forns index	0.493	<0.001 ***	0.201	0.019 *
SSAO activity	0.535	<0.001 ***		

**Table 3:** Linear regression analysis for independent predictors of liver stiffness. All variables correlating with liver stiffness are included in the model. We determined the regression coefficients with standard error, the beta coefficient and the statistics for co-linearity (tolerance and variance inflation factor (VIF)). Statistical outcome: *R* = 0.855, *R*^2^ = 0.730, significance < 0.001 ***. Gamma-glutamytransferase (GGT) was the strongest significant influencing variable for liver stiffness followed by VAP-1. Significance is given as * *p* < 0.05, and *** *p* < 0.001.

	Coefficient of Regression B	Standard Error	Beta	T Score	Significance	Tolerance	Variance Inflation Factor (VIF)
Constant	−7.096	11.249		−0.631	0.530		
VAP-1 conc.	0.012	0.006	0.185	2.218	0.029 *	0.414	2.414
SSAO activity	0.057	0.081	0.051	0.698	0.487	0.542	1.844
Age	0.080	0.125	0.066	0.637	0.526	0.270	3.697
Weight	0.062	0.056	0.065	1.103	0.273	0.822	1.217
APRI	1.362	2.529	0.084	0.539	0.591	0.118	8.465
Forns index	−0.689	1.176	−0.104	−0.586	0.559	0.091	10.932
GGT	0.047	0.008	0.474	6.091	0.000 ***	0.473	2.115
GPT	0.001	0.026	0.006	0.058	0.954	0.256	3.905
GOT	0.009	0.059	0.029	0.148	0.882	0.075	13.335
Bilirubin	−0.117	1.221	−0.007	−0.096	0.924	0.621	1.611
Cholesterol	0.008	0.018	0.027	0.432	0.667	0.743	1.345
Thrombocytes	−0.028	0.023	−0.147	−1.222	0.225	0.198	5.048
Endoglin	0.270	0.110	0.181	2.467	0.015 *	0.536	1.867
HsTnT (high-sensitivity troponin T)	−0.039	0.043	−0.058	−0.924	0.358	0.741	1.350
sFlt1 (soluble fms-like tyrosine kinase-1)	−0.017	0.060	−0.026	−0.275	0.784	0.308	3.243
PLGF (placental growth factor)	0.185	0.149	0.126	1.241	0.218	0.279	3.586
GDF15 (growth/differentiation factor 15)	0.000	0.001	0.040	0.313	0.755	0.174	5.747
HGF (hepatocyte growth factor)	0.001	0.001	0.140	1.454	0.149	0.308	3.251
proBNP (pro brain natriuretic peptid)	−0.001	0.000	−0.204	−1.873	0.064 *	0.243	4.118

## Supplementary Materials

The supplementary materials are available online at http://www.mdpi.com/2077-0383/8/1/103/s1.
