# Supplementary material for: Increased Circulating VAP-1 Levels Are Associated with Liver Fibrosis in Chronic Hepatitis C Infection
Source: J Clin Med. 2019 Jan 17;8(1):103. doi: 10.3390/jcm8010103 (PMC6352124; doi:10.3390/jcm8010103)
Supplement: Supplementary file 1 [file jcm-08-00103-s001.pdf]

Journal of Clinical Medicine

Supplementary Material

Increased Circulating VAP-1 Levels are Associated with Liver Fibrosis in Chronic Hepatitis C Infection

Marcel Kraemer, Marcin Krawczyk, Fozia Noor, Frank Grünhage, Frank Lammert and Jochen G. Schneider

Supplementary Figures

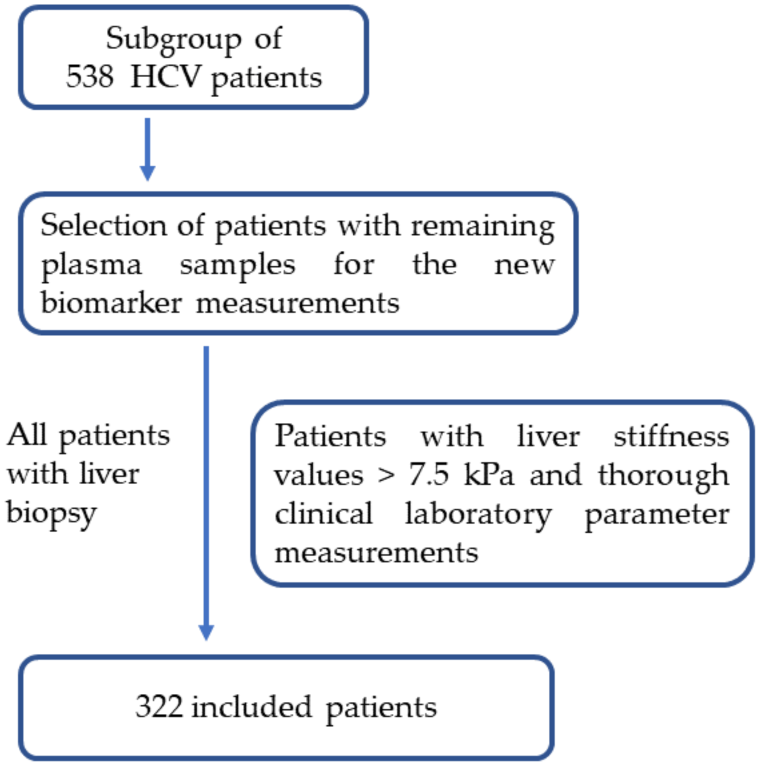

Figure S1. Description of the study cohort. The study was on a subgroup of a large cohort of 899 patients [15]. We selected 538 patients with chronic HCV infection. Of these patients 322 were selected based on the availability of blood samples, biopsy results, comprehensive measurement of clinical laboratory values, availability of liver stiffness values higher than 7.5 kPa to include patients representing fibrosis stage 2-4. Patients in the F0/F1 stage still represented the majority (153 out of 322).

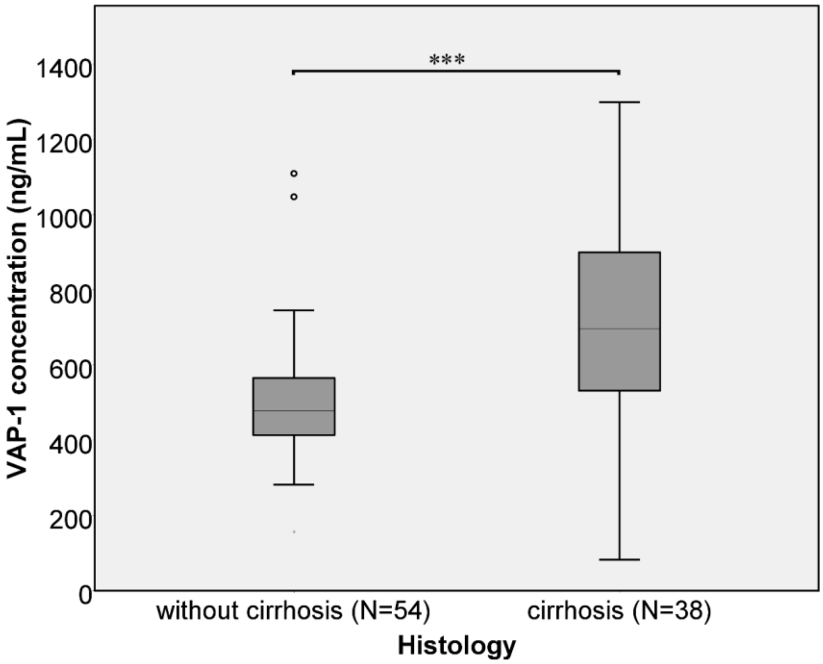

Figure S2. VAP-1 concentration without and with liver cirrhosis. The classification is based on the biopsy results (F0- F3 = no cirrhosis; F4 = cirrhosis; $p < 0.001$***). Significance is given as *** $p<0.001$.
